# Crossing the Hands Increases Illusory Self-Touch

**DOI:** 10.1371/journal.pone.0094008

**Published:** 2014-04-03

**Authors:** Polona Pozeg, Giulio Rognini, Roy Salomon, Olaf Blanke

**Affiliations:** 1 Center for Neuroprosthetics, School of Life Science, Ecole Polytechnique Fédérale de Lausanne, Lausanne, Switzerland; 2 Laboratory of Cognitive Neuroscience, Brain Mind Institute, School of Life Science, Ecole Polytechnique Fédérale de Lausanne, Lausanne, Switzerland; 3 Robotic Systems Laboratory 1, Institute of Microengineering, School of Engineering, Ecole Polytechnique Fédérale de Lausanne, Lausanne, Switzerland; 4 Department of Neurology, University Hospital, Geneva, Switzerland; University of Milan, Italy

## Abstract

Manipulation of hand posture, such as crossing the hands, has been frequently used to study how the body and its immediately surrounding space are represented in the brain. Abundant data show that crossed arms posture impairs remapping of tactile stimuli from somatotopic to external space reference frame and deteriorates performance on several tactile processing tasks. Here we investigated how impaired tactile remapping affects the illusory self-touch, induced by the non-visual variant of the rubber hand illusion (RHI) paradigm. In this paradigm blindfolded participants (Experiment 1) had their hands either uncrossed or crossed over the body midline. The strength of illusory self-touch was measured with questionnaire ratings and proprioceptive drift. Our results showed that, during synchronous tactile stimulation, the strength of illusory self-touch increased when hands were crossed compared to the uncrossed posture. Follow-up experiments showed that the increase in illusion strength was not related to unfamiliar hand position (Experiment 2) and that it was equally strengthened regardless of where in the peripersonal space the hands were crossed (Experiment 3). However, while the boosting effect of crossing the hands was evident from subjective ratings, the proprioceptive drift was not modulated by crossed posture. Finally, in contrast to the illusion increase in the non-visual RHI, the crossed hand postures did not alter illusory ownership or proprioceptive drift in the classical, visuo-tactile version of RHI (Experiment 4). We argue that the increase in illusory self-touch is related to misalignment of somatotopic and external reference frames and consequently inadequate tactile-proprioceptive integration, leading to re-weighting of the tactile and proprioceptive signals.The present study not only shows that illusory self-touch can be induced by crossing the hands, but importantly, that this posture is associated with a stronger illusion.

## Introduction

The skin defines the boundary of the organism and, as the largest human sensory organ, provides the most extensive interface with the environment through the tactile modality. Tactile information is also integrated with proprioceptive, visual, vestibular and auditory cues to construct multisensory representation of the body [1]–[4] and to generate the subjective experience of the body as one's own, i.e. body ownership [5]–[8]. Sense of body ownership also depends on the integration of motor signals [9]–[12], which by interaction with tactile perception, as in the case of self-touch, contributes to the self-awareness [13].

Localization of a tactile stimulus within external spatial coordinates comprises the location of the tactile cue on the body surface and its integration with proprioceptive signals [14]. These two processes are functionally and anatomically separated, relying on distinct neural mechanisms [15]–[18]. The tactile stimulus is first encoded with respect to a specific location on the skin (somatotopy) and processed by tactile neurons that have tactile receptive fields of varying size and location [19]–[22]. Then, in order to localize the touch in the external space, the tactile sensation is integrated with proprioceptive information about the current body position, as well as with the external signals from the visual and auditory system, and mapped into the common, external reference frame [14]–[16], [23], [24].

Several studies revealed that, when limbs are crossed, the integration of tactile with proprioceptive signals is hindered and localization of touch becomes less accurate [14], [25]. For example, accuracy of temporal order judgments (TOJ; of two successive tactile stimuli applied to each hand) drastically decreases if arms are crossed (as compared to uncrossed arms posture) and may even lead to the inversion of temporal order judgments [15]. Related findings have been observed in a spatial stimulus-response compatibility task [26], covert attention tasks [27] and crossmodal congruency effect tasks [28], [29].

However, little work has been done to study whether such “crossed hand effects” extend to the field of body ownership. An extensively used experimental protocol to manipulate hand ownership, generating the self-attribution of a fake hand via multisensory conflicts, is the rubber hand illusion (RHI) paradigm (the term *visual RHI* will be used further throughout the text). After observing a rubber hand that is placed next to and stroked in synchrony with one's own hand, hidden from view, participants report illusory self-attribution of the rubber hand. In this case, visual input dominates proprioceptive signals, inducing illusory sense of hand ownership for the fake hand [30]–[32]. The most common measures used to assess the illusion are questionnaire ratings and proprioceptive drift, i.e. shift of proprioceptively perceived location of one's own hand towards the rubber hand [30], [31], [33]–[36]. Importantly, illusory ownership decreases with larger visuo-proprioceptive spatial separations [32], [37], [38]. The illusion also decreases with lessened resemblance of the stroked object to a hand shape [36], [39], and different handedness of the fake arm [35], [36], [38], [40].

**Figure 1:**
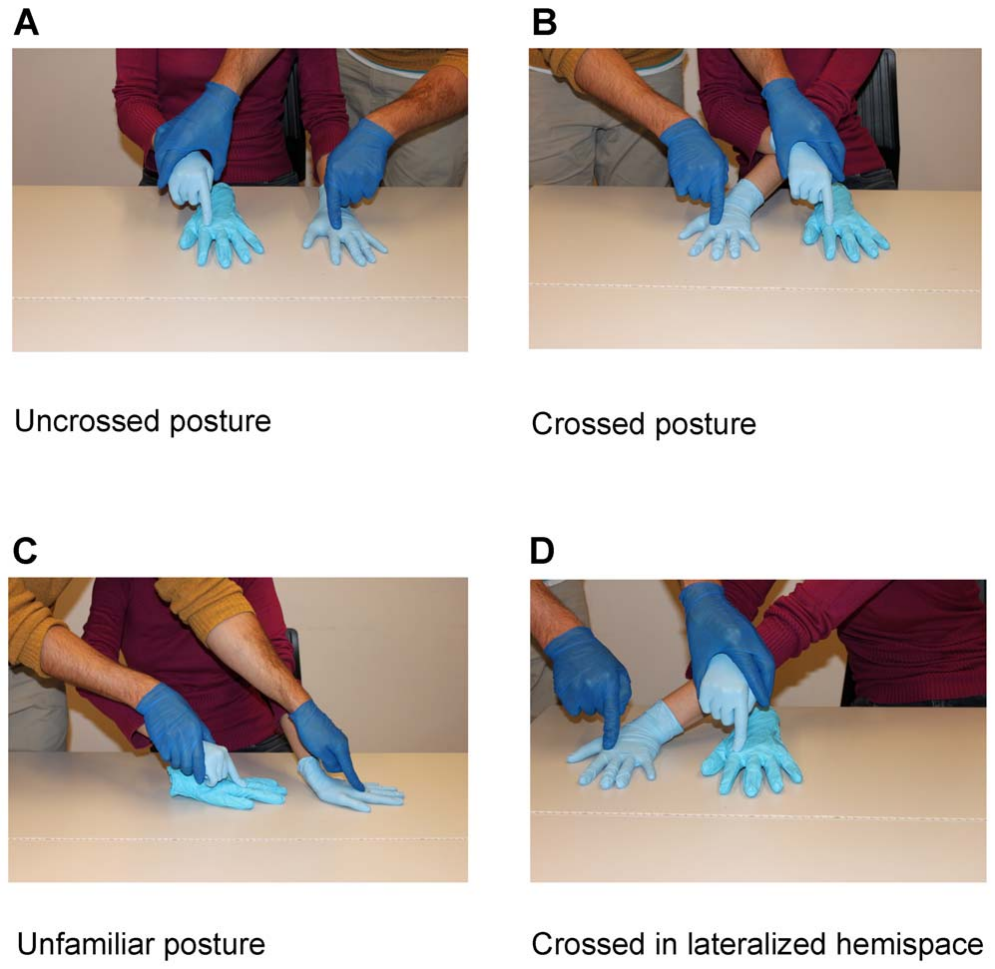
Hand postures in the tactile RHI (Experiments 1–3). (A) *Uncrossed posture*: The rubber hand (middle finger) is aligned with the participant's body midline axis. The participant's left hand rests in the left hemispace, 20 cm away from the rubber hand (distance between both middle fingers). (B) *Crossed posture*: The rubber hand is aligned with the participant's body midline axis. The participant's left hand is crossed over the body midline and rests in the participant's right hemispace, 20 cm away from the rubber hand (distance between the middle fingers). (C) *Unfamiliar posture*: The rubber hand is rotated by 90 degrees to the participant's left; its MCP joint of the middle finger is aligned with the participant's body midline. The participant's left hand rests in his left hemispace and is turned in the same direction as the rubber hand. The distance between the MCP joint of the participant's left middle finger and the rubber hand's middle finger MCP is 20 cm. (D) *Crossed in lateralized hemispace*: The rubber hand is positioned in the participant's right hemispace, with the distance of 20 cm between the rubber hand middle finger and the participant's body midline. The participant's left hand is crossed under his right arm and rests in the right hemispace, 20 cm to the right of the rubber hand (distance between the middle fingers).

In the tactile, non-visual variant of RHI [41] (the term *tactile RHI* will be used further throughout the text), the RHI paradigm is modified, so that the experimenter moves the index finger of a blindfolded participant to stroke a rubber hand, while he strokes - at the same time - the corresponding part of the participant's other hand (see Figure 1). Synchronized stroking induces illusory self-touch, i.e. the illusion of touching one's own hand, while instead one is physically touching the fake hand [33], [41]–[43].

We tested the tactile RHI paradigm in combination with a hand crossing manipulation in order to examine the effect of hand posture on the process of tactile-proprioceptive integration and induction of illusory self-touch (for a related example see [44]). We predicted that a “crossed hand effect” due to crossing of the hands during the tactile rubber hand illusion will modulate the strength of the tactile RHI. According to earlier observations [25], [28], [45] showing that crossing the hands impairs tactile-proprioceptive integration, such a posture manipulation may result in a decreased illusion. Alternatively, as impaired tactile-proprioceptive integration hinders the ability to localize tactile stimuli on one's own body, and therefore interferes with “standard” multisensory body representations, crossing the hands may lead to the increase of illusory self-touch. Such potential boosting of the illusion would be in itself novel finding because other postural manipulations have been shown so far to decrease the RHI effect.

We first report the results of three consecutive experiments in which we manipulated hand posture while inducing illusory self-touch in the tactile RHI. In Experiment 1 we explored the effect of crossing the hands on illusory self-touch and proprioceptive drift in the tactile RHI paradigm. The results confirmed the second hypothesis that crossing the hands across the body midline increased illusory self-touch as compared to uncrossed posture in the tactile RHI. However, crossing the hands did not modulate proprioceptive drift as compared to uncrossed hands posture. We next investigated whether the increase in the tactile RHI depends on the familiarity of the posture manipulation. Therefore we compared the strength of the illusory self-touch when participants had their hands in a standard uncrossed posture and when they were in an unfamiliar posture, i.e. with their left hand placed in the left hemispace and rotated by 90 degrees to the left (Experiment 2). Based on the evidence that hand position may not be only coded with respect to the body midline, but also in relation to the other hand [46]–[48], we further tested whether the increase in the tactile RHI is specific to crossing the body midline axis, or generalized to any crossing hands postures, independently from where they are placed in space (Experiment 3). Hence participants were presented with the tactile RHI paradigm while they kept their hands crossed across their midline or within their right hemispace. We found that the increase in the strength of the tactile RHI was not related to the unfamiliarity of the hand position (Experiment 2) and that the illusory self-touch was equally strengthened regardless of where in the peripersonal space the hands were crossed (Experiment 3). Finally, in Experiment 4, we explored whether the boosting effect of the crossed hand posture also applies to illusory hand ownership and proprioceptive drift in the visual RHI paradigm. Based on extensive evidence regarding the dominant role of vision over proprioception in estimating hand position and localizing tactile stimuli [49]–[52], we hypothesized that crossing the hands would not significantly affect the intensity of the illusory ownership in the visual RHI paradigm.

## Materials and Methods

All participants were recruited by an advertisement on the EPFL campus (École Polytechnique Fédérale de Lausanne, Switzerland). They were fluent in English, right-handed and had normal touch perception as assessed by self-report. Each participant only took part in one experiment. All participants were naive to the purpose of the study and gave written informed consent to take part in the study. The study was approved by the local ethics committee (La Commission d’Ethique de la Recherche Clinique de la Faculté et de Medicine de l’Université de Lausanne) and was conducted according to the ethical standards laid down in the Declaration of Helsinki. Participants were reimbursed for their participation in the study with 10 CHF.

### Experiment 1: Effect of crossing on illusory self-touch

#### Participants

14 participants (1 female) participated in Experiment 1. Their age ranged from 22 to 35 years (M = 26.4 years, SD = 3.9 years).

#### Experimental design and procedure

We employed the tactile-proprioceptive paradigm from Ehrsson et al. [41] to induce illusory self-touch. The participant was seated behind a desk, wearing a blindfold to prevent any visual input and plastic gloves to match the tactile sensation of the rubber hand. The experimenter was stroking a gloved left rubber hand with the participant's right index finger while at the same time stroking the participant's left hand (Figure 1, part A). The left rubber hand was aligned with the participant's body midsagittal plane. The experimental design contained 2 within-subject factors: synchrony (asynchronous versus synchronous tactile stimulation) and hand posture (uncrossed versus crossed posture). The tactile stimulation of both hands, composed of alternating strokes and taps, was temporally and spatially matched in the synchronous conditions and unmatched in the asynchronous conditions. Note that in this tactile version of the rubber hand illusion there is a tactile-proprioceptive mismatch between the proprioceptive position of the passively stroking hand (touch cue at the stroked tip) and the proprioceptive position of the stroked hand (touch cue at the stroked hand; see Figure 1).

In the “uncrossed posture” condition, the participant's left hand rested in the participant's left hemispace, palm turned downwards with the middle finger being 20 cm from the body midline axis. A left dummy rubber hand was aligned with the body central sagittal plane. In the “crossed posture” condition, the participant's left hand crossed the body midline and rested in the right hemispace, again 20 cm from the body midline axis. The order of four conditions was randomized across participants. The tactile stimulation in each condition lasted for 60 seconds. Before and immediately after each condition the participant was asked to indicate the location of his left hand. For this we asked him to place his right middle finger above his left middle finger, without making any contact between them. The position of the right middle finger was recorded. The proprioceptive drift was defined as the difference between the pre- and post-stimulation measures. After each condition, the participant was also asked to answer the three-item questionnaire adapted from Ehrsson et al. [41]. The first item referred to illusory self-touch (*I felt like I was touching my hand*), while the other two served as control items for suggestibility (*I felt like I had another hand; I felt like my left hand was moving*). Here was asked to indicate on the 7-point Likert scale the intensity of subjective feeling described in each item (0  =  not experienced at all, 6  =  strongly experienced).

### Experiment 2: Effect of unfamiliar posture on illusory self-touch

#### Participants

14 (2 females) participants were involved in Experiment 2. Their age ranged between 24 and 29 years (M = 25.1 years, SD = 2.1 years).

#### Experimental design and procedure

In Experiment 2 we investigated whether the strength of illusory self-touch in the tactile rubber hand illusion was related to the unfamiliar posture of the hands in the crossed position. The same experimental design and procedure was used as in Experiment 1; however, instead of the crossed posture condition, we included an unfamiliar posture condition in the design and compared it with illusory self-touch in the uncrossed posture condition. In the “unfamiliar posture” conditions, the participant's left hand was placed on the table (in the left hemispace) and rotated by 90 degrees to the left. The rubber hand was turned in the same direction and rested on the midline axis, so the distance between the middle fingers' metacarpophalangeal (MCP) joints of the real and rubber hand was 20 cm (Figure 1, part B). Again, all tactile stimulations lasted for 60 seconds and the order of the four conditions was randomized across subjects. The subjective reports and the measure of proprioceptive drift were obtained in the same manner as in Experiment 1.

### Experiment 3: Effect of crossing in lateralized hemispace on illusory self-touch

#### Participants

15 participants (7 females) took part in Experiment 3. Their age ranged between 18 and 34 years (M = 24.2, SD = 4.1).

#### Experimental design and procedure

In Experiment 3 we investigated whether the increase in the illusory self-touch when hands were crossed was caused by crossing the body midline and thus positioning hands in the opposite hemispace or to crossing the hands per se (within the same hemispace for example). As in Experiment 1 two factors (synchrony and hand posture) were manipulated. The hand posture factor included “crossed posture” and “crossed in lateralized hemispace” conditions. The settings of the former are described in Experiment 1. In the “crossed in lateralized hemispace” condition, hands were crossed in the participant's right hemispace. The left hand was positioned 50 cm (the distance from the tip of the middle finger) from the body midline axis; while the rubber hand rested 30 cm away from the body midline axis in the same, right hemispace. The distance between the rubber and the stroked hand's middle finger was again 20 cm (Figure 1, part C). The experimental procedure and the outcome measures were the same as in Experiment 1.

### Experiment 4: Effect of crossing in the visual RHI

In Experiment 4 we explored whether crossing the hands would affect illusory hand ownership and proprioceptive drift in the visual RHI paradigm [30].

#### Participants

14 participants (5 females) were participating in Experiment 4. Their age ranged between 21 and 29 years (M = 23.8, SD = 2.26).

#### Experimental design and procedure

A setup similar to the one described in Tsakiris & Haggard [36] was used and has been described previously to successfully induce the rubber hand illusion [53]. It consisted of a black wooden frame (100×50 cm), which was put on a desk in front of a participant and covered by a two-way mirror 23 cm above the desk. To occlude the sight of the participant's hands, a black paper was put under the mirror, leaving the middle third of the surface open to enable the view on the right rubber hand, which was placed in the centre of the wooden frame, aligned with the participant's body midline axis. A black fabric was installed inside the frame to occlude any side view of the participant's hands and forearms. Due to the two-way mirror the participant was able to see the rubber hand during tactile stimulation when the lights in the frame were turned on. During the proprioceptive judgment task, the rubber hand was hidden by putting the lights in the frame off, and a ruler on the top of the mirror was shown.

**Figure 2:**
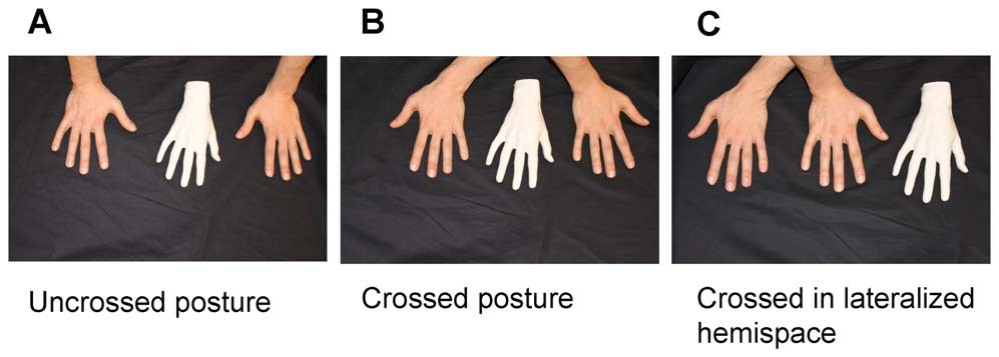
Hand postures in the visual RHI (Experiment 4). (A) *Uncrossed posture*: The rubber hand was positioned palm downwards and aligned (middle finger of the rubber hand) with the participant's body midline. The participant's hands were in their anatomical position each resting in its corresponding hemispace, 20 cm from the rubber hand (distance between the middle fingers). (B) *Crossed posture*: The rubber hand was aligned with the participant's body midline axis. The participant's right hand was crossed over the left one. Both hands rested on the desk, each with the distance of 20 cm to the rubber hand (distance between the middle fingers). (C) *Crossed in lateralized hemispace*: The rubber hand was again aligned with the participant's body midline axis. His left hand was crossed under his right arm in his right hemispace. The distance of 20 cm was kept between the rubber hand and the right hand middle fingers and 40 cm between the rubber hand and the left hand middle fingers.

The experimenter placed the participant's hands inside the wooden frame. A right rubber hand was placed and aligned with the subject's midsagittal axis. The position of the hands was fixed depending on the experimental condition. In the “uncrossed posture” condition, the participant's hands were laid down in the anatomical position, with 40 cm of distance between both middle fingers. In the “crossed posture” the right hand was crossed over the left one, again, keeping 40 cm between both middle fingers. In the “crossed in lateralized hemispace” condition the participant's left hand was crossed under his right arm in his right hemispace. The same distance of 20 cm was kept between the rubber hand and the right hand middle fingers across all three conditions (Figure 2). In all conditions, the experimenter synchronously stroked and tapped the participant's right hand and the rubber hand. The latter was always in the same anatomical position as was the participant's stroked hand. However, depending on the condition it was not always positioned in the same hemispace. The order in which the three conditions were presented was randomized across participants. Before and after each condition, the participant was asked to make a proprioceptive judgment by verbally indicating on the ruler the perceived location of his right middle finger, while the hands were occluded from his vision. Rulers with a different onset were used for each proprioceptive judgment to prevent the participant from repeating the same value over the trials. After each condition, participants filled out the 9-item Visual Rubber Hand Illusion questionnaire, adapted from [30].

### Data analysis

Questionnaire scores in Experiment 1, 2 and 3 significantly deviated from normal distribution (Shapiro-Wilk test of normality), therefore they were analysed with non-parametric statistical tests. First, the data sets were analysed with Friedman's ANOVA, and if significant, they were followed up with pair-wise comparisons, using the 2-tailed Wilcoxon's signed rank test. Three planned comparisons were made for each data set in the tactile RHI experiments, where the ratings of the two synchronous conditions were compared with their respective asynchronous pair, and those of the two synchronous, but different posture conditions, with each other. The p-values were corrected for multiple comparisons using the Bonferroni method, where α(corrected) = .05/3 = .0167. The data acquired from the questionnaire ratings in Experiment 4 and proprioceptive drift measurements from all 4 experiments were analysed with repeated measures analysis of variance (ANOVA) and when required followed-up with two-tailed paired sample t-tests.

## Results

### Experiment 1: Effect of crossing on illusory self-touch

**Figure 3:**
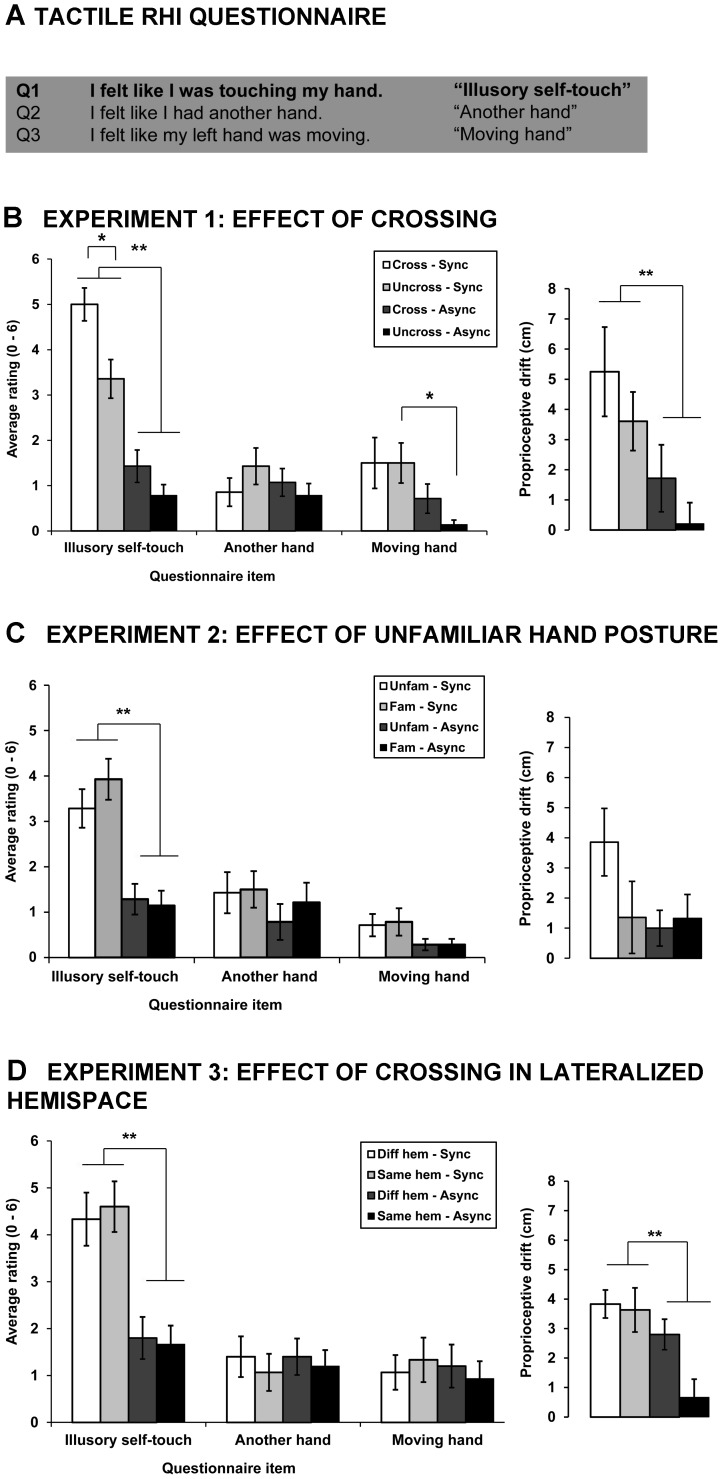
Questionnaire scores and proprioceptive drift results in the tactile RHI. (A) Questionnaire items adapted from [41] used in the Experiments 1 – 3. (B) Average questionnaire ratings and proprioceptive drift in Experiment 1. The participants reported stronger illusion in the synchronous as compared to asynchronous conditions. The illusion strength in the synchronous condition was enhanced when the hands were crossed as compared when uncrossed.Larger drift was observed in synchronous conditions.(C) Average questionnaire ratings and proprioceptive drift in Experiment 2. The participants reported stronger illusion in the synchronous conditions; however, no difference in the illusion strength was found between the familiar and unfamiliar hand posture. The proprioceptive drift did not significantly differ across the four conditions. (D) Average questionnaire ratings and proprioceptive drift in Experiment 3. The participants reported stronger illusion in the synchronous conditions; however, no difference in the illusion ratings were found between the synchronous conditions when hands were crossed over midline and when they were crossed in lateralized hemispace. The synchrony of stroking as well as crossing the hands over midline significantly increased the proprioceptive drift. The error bars depict the standard error of the mean. Sync  =  synchronous, Async  =  asynchronous, Cross  =  crossed, Uncross  =  uncrossed, Fam  =  familiar, Unfam  =  unfamiliar, Diff hem  =  crossed over midline, Same hem  =  crossed in lateralized hemispace.

In Experiment 1 we explored how crossing the hands over the body midline affects illusory self-touch. Statistical analysis of the subjective ratings revealed that the reported strength of illusory self-touch (Item 1: *I felt like I was touching my hand*) significantly differed across the four conditions (χ^2^(3) = 36.02, p<.001). Using the adjusted α level of .0167 the follow-up Wilcoxon signed rank test revealed that participants rated the experience of self-touch stronger when the stroking was synchronous in uncrossed (M = 3.36, SD = 1.60; Z = −3.071, p = .002, r = .580) as well as in crossed hand postures (M = 5.00, SD = 1.36; Z = −3.320, p = .001, r = .627) as compared to asynchronous stroking (uncrossed: M = 0.79, SD = 0.89; crossed: M = 1.43, SD = 1.34). Importantly, having the hands crossed during synchronous tactile stimulation significantly increased the ratings of illusory self-touch as compared to the uncrossed posture condition (Z = −2.700, p = .007, r = .510). The observed increase in the illusion strength when hands were crossed was robust as 79% of participants rated the illusory self-touch at 4 or higher (compared to only 50% in uncrossed condition; χ^2^ test: p = .033) (see Figure 3).

We further found that the mean of the illusory touch ratings after synchronous stimulation was significantly higher (adjusted α level = .0167) than the mean ratings on both control items in the crossed (Item1/Item2: Z = −3.325, p = .001, r = .628; Item1/Item3: Z =  = 3.204, p = .001, r = .606) and uncrossed hand postures (Item1/Item2: Z = −2.988, p = .003, r = .565; Item1/Item3: Z = −2.692, p = .007, r = .509). The average ratings of the control Item 2 (*I felt like I had another hand*) did not significantly differ across the four conditions (χ^2^(3) = 0.953, p = .813). Significant differences in ratings were found for the control Item 3 (*I felt like my left hand is moving*) (χ^2^(3) = 11.077, p = 0.011). The planned post-hoc comparisons with the adjusted α level of .0167 revealed significantly higher ratings of the item in the uncrossed-synchronous conditions as compared to the uncrossed-asynchronous condition (Uncross Sync/Uncross Async: Z = −2.536, p = .011; Cross Sync/Cross Async: Z = −1.361, p = .174; Cross Sync/Uncross Sync: Z = −.000, p = 1.000). Taking into account the significant synchrony modulation of the Item 3 ratings, its use as a control item should be taken into consideration.

Drift analysis showed that the proprioceptive drift of the stimulated hand was greater in the synchronous versus asynchronous conditions (F(1,13) = 10.365, p = .007, η_p_^2^ = 0.444). No significant main effect of hand posture on the proprioceptive drift (F(1,13) = 1.833, p = .199, η_p_^2^ = 0.124) nor interaction between the synchrony of stroking and hand posture (F(1,13) = .005, p = .945, η_p_^2^ = 0.000) were observed.

### Experiment 2: Effect of unfamiliar posture on illusory self-touch

In Experiment 2 we tested whether the increase in illusory self-touch is due to the unfamiliar posture rather than to the crossing of the hands. Friedmann's ANOVA showed significant differences between the mean ratings of the four conditions (χ^2^(3) = 30.487, p<.0001). Post-hoc comparisons with adjusted α level of .0167 revealed that the participants rated illusory self-touch as more intense when the tactile stimulation was synchronous in familiar (M = 3.93, SD = 1.69) as well as in unfamiliar conditions (M = 3.29, SD = 1.59) as compared to asynchronous stroking (familiar: M = 1.14, SD = 1.23, Z = −3.104, p = .002, r = .587; unfamiliar: M = 1.29, SD = 1.27, Z = −3.089, p = .002, r = .584). Moreover, the illusion intensity in familiar and unfamiliar postures when the stroking was synchronized did not significantly differ (Z = −1.809, p = .070, r = .342), showing that the “crossed hands effect” on illusory self-touch does not depend on the familiarity of the posture. The between-subject comparison of the illusory self-touch ratings in the unfamiliar – synchronous condition with the ratings in the crossed – synchronous condition (Experiment 1) showed the latter to be significantly higher (Mann-Whitney U test: Z = −2.743, p = .006, r = .518). This comparison further indicated that the increase in the illusory self-touch was specific for crossed hand posture. The average ratings of the other two control items were low (M≤1.5, SD<1.70). The statistical analysis showed that the ratings of Item 2 did not significantly differ across the four conditions (χ^2^(3) = 6.000, p = .112) whereas significant differences were found in the ratings for the Item 3 (χ^2^(3) = 8.864, p = .031); however, none of the planned comparisons using the adjusted α level of .0167 yielded significant differences (Familiar Sync/Familiar Async: Z = −2.121, p = .034, r = .401; Unfamiliar Sync/Unfamiliar Async: Z = −1.656, p = .098, r = .313; Unfamiliar Sync/Familiar Sync: Z = −0.378, p = .705, r = .071).

No significant main effect of synchrony (F(1,13) = 1.72, p = .212, η_p_^2^ = 0.117), hand posture (F(1,13) = 2.30, p = .153, η_p_^2^ = 0.150) nor interaction (F(1,13) =  3.73, p = .076, η_p_^2^ = 0.223) was found on the proprioceptive drift toward the rubber hand.

### Experiment 3: Effect of crossing in lateralized hemispace on illusory self-touch

In Experiment 3 we explored whether the increase in illusory self-touch was specific to the fact that hands crossed the body midline or whether the increase was caused by crossing of the hands per se (without crossing the body midline). The illusory self-touch ratings significantly differed across the four conditions (χ^2^(3) = 24.891, p<.0001). Post-hoc analyses with adjusted α level of .0167 revealed that, again, illusory self-touch was reported as more intense when the applied tactile stimulation was synchronous (crossed over the midline: M = 4.33, SD = 2.19; crossed in lateralized hemispace: M = 4.60, SD = 2.09) as compared to the asynchronous conditions (crossed over the midline: M = 1.80, SD = 1.74, Z = −2.767, p = .006, r = .505; crossed in lateralized hemispace: M = 1.67, SD = 1.54, Z = −3.234, p = .001, r = .591). Importantly, the intensity of illusory self-touch did not differ depending on where in peripersonal space the hands were crossed (Z = −0.516, p = .606, r = .094). The average ratings of the other two control items were low (M<1.5, SD<1.85) and did not significantly differ across the four conditions (Item 2: χ^2^(3) = 0.953, p = .813; Item 3: χ^2^(3) = 0.395, p = . 941).

The between-subject comparison of the self-touch illusory item ratings in the crossed in lateralized hemispace-synchronous condition with the ratings in the uncrossed – synchronous condition in Experiment 1 showed the crossed in lateralized hemispace condition to be significantly higher (Mann-Whitney U test: Z = 2.242, p = .025, r = .423). The ratings in this condition were also significantly higher from the unfamiliar-synchronous condition in Experiment 2 (Mann-Whitney U test: Z = −2.346, p = .019, r = 0.436).

The participants made larger pointing errors towards the rubber hand after they had been synchronously stroked compared to the conditions of asynchronous tactile stimulation (F(1,14) = 12.07, p = .004, η_p_^2^ = 0.463). The arm posture also significantly modulated the proprioceptive drift, which was larger in the conditions where arms were crossed over the body midline axis (F(1,14) = 4.71, p = .048, η_p_^2^ = 0.252). No interaction effect was found between synchrony of stimulation and the position of the crossed hands (F(1,14) = 1.63, p = .222, η_p_^2^ = 0.105) (see Figure 3).

### Experiment 4: Effect of crossing in the visual RHI

When the standard visual RHI paradigm was administered, synchronous stroking in all three hand postures successfully induced illusory ownership (uncrossed hands: M = 4.50, SD = 1.13; crossed over midline: M = 4.88, SD = 1.04; crossed in lateralized hemispace: M = 4.43, SD = 1.41) that significantly differed from the control items (uncrossed hands: M =  2.26, SD = 0.98, t(13) = 5.873, p = .0001; crossed over midline: M = 2.40, SD = 1.12, t(13) = 8.144, p<.0001; crossed in lateralized hemispace: M = 2.32, SD = 0.95, t(13) = 5.759, p<.0001). However, no differences in mean ratings of any of the questions were found between different hand posture conditions (Q1 (*It seemed as if I were feeling the touch in the location where I saw the rubber hand touched*): F(2,12) = 1.591, p = .244, η_p_^2^ = 0.210; Q2 (*I felt as if the rubber hand were my hand*): F(2,12) = 0.668, p = .531, η_p_^2^ = 0.100; Q3 (*It seemed as though the touch I felt was caused by the experimenter touching the rubber hand*): F(2,12) = 0.847, p = .453, η_p_^2^ = 0.124; Q4 (*It felt as if my (real) hand were drifting towards the rubber hand*): F(2,12) = 0.127, p = .882, η_p_^2^ = 0.021; Q5 (*It seemed as if I might have more than one right hand or arm*): F(2,12) = 0.469, p = .637, η_p_^2^ = 0.072; Q6 (*It seemed as if the touch I was feeling came from somewhere between my own hand and the rubber hand*): F(2,12) = 0.209, p = .815, η_p_^2^ = 0.034; Q7 (*It felt as if my (real) hand were turning ‘rubbery’*): F(2,12) = 0.427, p = .662, η_p_^2^ = 0.066; Q8 (*It appeared (visually) as if the rubber hand were drifting towards my hand*): F(2,12) = 0.777, p = .481, η_p_^2^ = 0.115; Q9 (*The rubber hand began to resemble my own (real) hand, in terms of shape, skin tone, freckles or some other visual feature*): F(2,12) = 2.128, p = .162, η_p_^2^ = 0.262).

**Figure 4:**
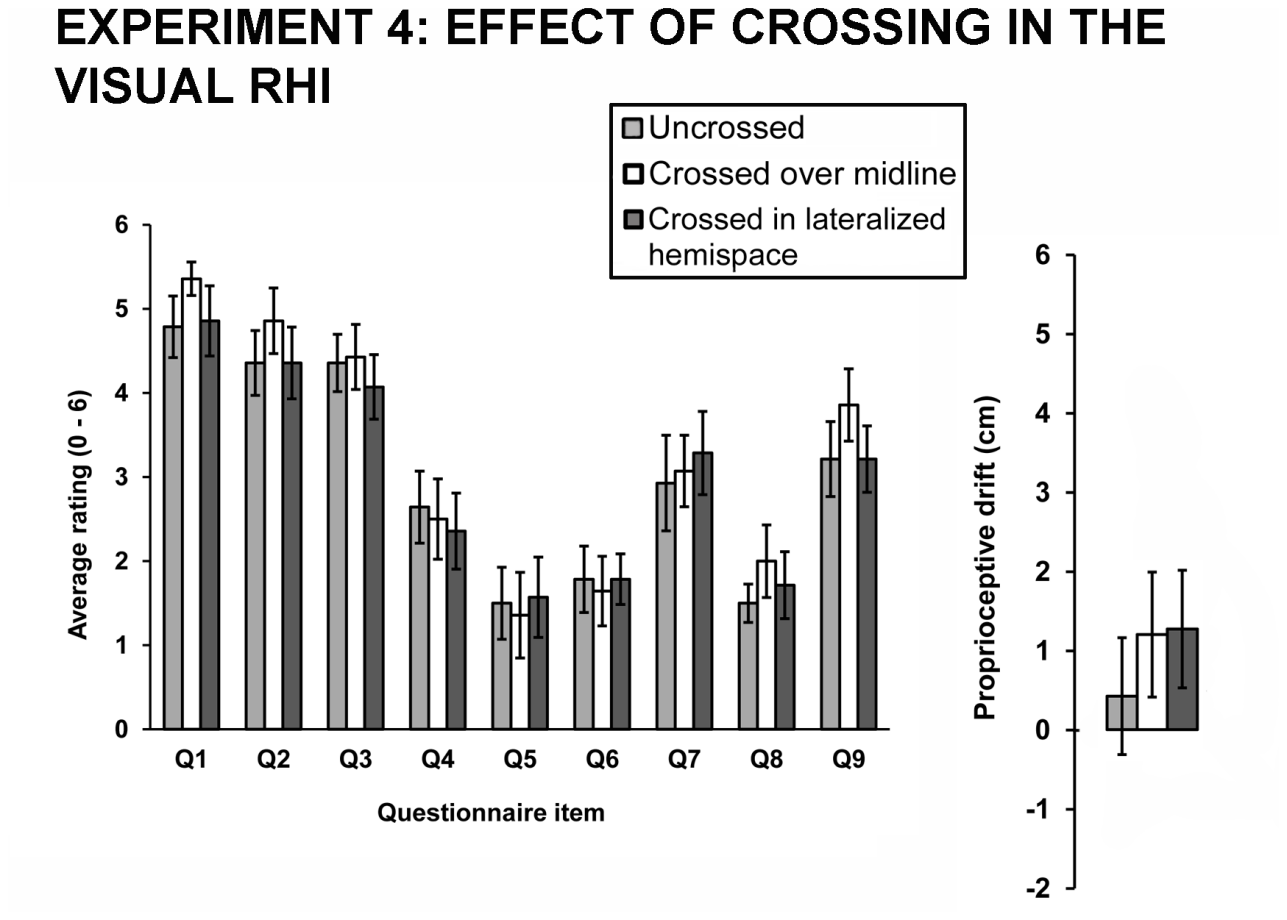
Questionnaire scores and proprioceptive drift results in the visual RHI. *Left panel* showing the average ratings of the questionnaire items for three different hand postures in the visual RHI paradigm (Experiment 4). The average ratings indicate that participants experienced the illusion (first three items). However, the posture manipulations did not affect the intensity of the illusion. The error bars represent the standard error of the mean. *Right pane*l showing average proprioceptive drift measures in the visual RHI paradigm (Experiment 4) for the three hand postures. The differences between the three conditions did not reach the level of significance. The error bars represent the standard error of the mean.

There were no significant differences between the three conditions in the proprioceptive drift (F(2,12) = 0.712, p = .510, η_p_^2^ = 0.106). The results are shown in Figure 4.

## Discussion

In four experiments we examined the effect of hand posture in the tactile and visual RHI. We show, for the first time, that crossing the hands, while synchronous tactile stimulation is given, increases illusory self-touch, i.e. the illusory sensation that one is touching oneself while one's own index finger physically touches a rubber hand. Follow-up experiments showed that the increase in the illusion strength was not related to unfamiliar hand position (Experiment 2) and that illusory self-touch was equally strong regardless of where in the peripersonal space the hands were crossed (Experiment 3). These effects were evident from subjective reports, i.e. the questionnaire data, but not from proprioceptive judgments which were not modulated by crossing the hands. Finally, in contrast to the illusion increase in the tactile RHI, the crossed hand postures did not alter illusory hand ownership or proprioceptive judgments in the visual RHI (Experiment 4). Showing that the rubber hand illusion can be induced by manipulating tactile and proprioceptive input and its timing, the present data not only demonstrate that illusory self-touch can be induced by crossing the hands, but importantly, that this posture is associated with a stronger illusion.

To accurately localize a tactile event, tactile information coded within the somatotopic (skin surface anchored) coordinates is combined with proprioceptive and visual signals in a multisensory representation of the body [8], [17], [54], [55]. These hand representations are constantly updated as we move by available multisensory information, amongst which the visual modality is an especially reliable source and therefore strongly biases the remapping process [50], [51], [56].

In the tactile version of the RHI paradigm, no visual information about the position of the hand in space is available, and therefore location of touch in external reference frame depends on the combination of proprioceptive and tactile cues. However, tactile and proprioceptive cues from the two hands are ambiguous: subjects feel their left hand being touched while at the same time their right hand touches a rubber hand. The synchrony between the two tactile inputs suggests that they refer to the same object, therefore inducing illusory sensation of touching one's own hand instead of the rubber hand. However, such perceptual solution raises a conflict between tactile and proprioceptive signals because, in terms of proprioceptive information, the two tactile signals coming from the hands cannot pertain to the same percept. As a consequence, the incongruent proprioceptive signals between the stroking and stroked hands are overridden by the more probable interpretation that two tactile events (spatially separated) are occurring at a single external location [57], [58]. Consequently, the tactile-proprioceptive conflict is resolved in the experience of touching one's own hand, i.e. in illusory self-touch.

The new result from our study shows that postural manipulation i.e. crossing the hands has a boosting effect on the illusion. This finding diverges from previous studies, which have repeatedly shown that postural manipulations, other than having a hand in a default anatomical position and aligned with the rubber hand, lead to a decrease in the indices of the visual RHI [32], [37], [38]. A recent study also demonstrated that, in the tactile RHI, illusory self-touch decreases with increasing distance between the participant's stroked hand and the rubber hand and with increased incongruence in orientation between both hands [42].

The crossed hand related increase in the illusory self-touch can be explained by misalignment of somatotopic and external reference frames and consequently inadequate tactile-proprioceptive integration. In order to correctly localize the tactile event and act upon it, the somatotopic information is integrated with proprioceptive signals about the current position of hands and translated into a common, external space reference frame [25], [28], [45], [59]. Mapping tactile stimuli in the external, multisensory peripersonal space is an automatic process, developed through early sensory experiences, driven primarily by vision [60], [61]. The brain has a default way to map tactile stimuli from the somatotopic coordinates of the hand to its respective ipsilateral hemifield in the peripersonal space [15], [60]. As crossing the hands introduces a strong conflict between the somatotopic and external space coordinates, the tactile-proprioceptive integration and re- mapping of tactile stimuli into the external space are altered. The misalignment of proprioceptive and tactile reference frames induces re-weighting of the tactile and proprioceptive signals. Due to generally less reliable proprioceptive cues, the probability that two spatially separated but temporally matched tactile events are interpreted as occurring at a single external location during the tactile RHI increases. Consequently, the estimation of the hand position is recalibrated to match the resolution of the tactile-proprioceptive conflict. As a result, the illusory self-touch is experienced as stronger.

Previous evidence has shown deficits in tactile re-mapping when crossing the hands and a consequent loss of perceptual reliability of proprioceptive information. An example of the crossed hand related remapping impairment is the increased difficulty to mentally visualize an object when it is bimanually explored with crossed hands while being blindfolded [62]. Moreover, in the TOJ task, when a blindfolded participant judges the temporal order of two successive tactile stimuli applied to each hand, the performance accuracy drastically decreases when arms are crossed [15], [25], [63]. Shorter inter-stimuli intervals (<300 ms) even lead to subjective inversion of the temporal order [15]. The same crossing decrease in the TOJ performance has also been shown for crossed fingers [16]. The boosting effect of crossing hands in the tactile RHI can be related to the so-called Aristotle illusion. In this illusion, rubbing the external sides of two adjacent and crossed fingers with a spherical object produces a percept of touching two distinct objects [64]. In a similar manner, simultaneous tactile stimulation of the inner parts of crossed fingers induces a sensation of touching only one surface [65], [66].

The role of the remapping process in the perception of the tactile rubber hand illusion is also supported by studies in congenitally blind people. For example, it has been shown that blind people have smaller crossed hand effects in TOJ task [60] and, moreover, they do not experience illusory self-touch in the tactile RHI [58]. As suggested, congenitally or early blind people do not automatically remap somatotopic information into the external frame of reference, which is dominated by vision, but they rather rely on internal, anatomically based or egocentric reference frames [60]. Hence, in their case the automatic remapping in the external reference frame does not interfere with the tactile localization - as compared to sighted persons who mostly rely on the common external frame of reference, dominated by vision. However, the performance of sighted persons on the TOJ task improves when they perform the task having their hands crossed behind their back, that is in the peripersonal space not defined by visual input [67].

An alternative explanation for the increase of illusory self-touch is that proprioceptive cues of crossed hands increase the likelihood of single sensory event perception. When the hands are crossed, the angles of the upper arms are rotated towards each other, which is the position usually adopted when the hands are actually in tactile contact, compared to the angle when hands are positioned in parallel. The probability of self-touch under everyday conditions is thus higher when the hands are crossed, due to proprioceptive cues from the position of the arms (see also [42]).

In Experiment 2 we showed that the unfamiliar hand posture itself did not lead to the same boosting effect on the illusion as the crossed posture did, and it also did not decrease the ratings of the illusion when compared to the uncrossed posture. Moreover, Experiment 3 revealed that not only crossing the hands over the midline, but also crossing them in one hemispace, increases the ratings of the self-touch illusion. First, these findings suggest that the remapping impairments and the consequent increase of illusion are specific to the crossed posture. Secondly, the findings question the interpretation of self-touch illusion by White and Aimola Davies [42], who argue that the proprioceptive cues (coming from the elbow and shoulder rotation) contribute to the likelihood of perceiving two tactile stimuli as a single sensory event. In the unfamiliar posture the participant's left angular rotation of the shoulder joint was enhanced, whereas the left shoulder joints' rotation remained relatively the same as in the uncrossed posture. The proprioceptive incongruence between the participant's left and right hand was even more accentuated in Experiment 3, where the right hand (being crossed over the left) was positioned at the most extreme side of the participant's left hemispace. According to the interpretation of White and Aimola Davies these proprioceptive cues originating from the unfamiliar and crossed in a lateralized hemispace postures should decrease if not abolish the illusion. Nevertheless, the two explanations are not necessarily exclusive. Because proprioceptive signals have large variance and low reliability compared to visual information (at least in the frontal peripersonal space) [32], [49], [68], the illusory self-touch is experienced as long as the hands occupy a relatively limited and overlapping spatial range. When the distance between the hands increases, which is signalled by proprioceptive cues, the likelihood to experience two tactile stimuli as a single sensory event dissipates. In the present study, the distance between the two crossed hands (or two tactile stimuli) remained unchanged, but as the tactile-proprioceptive integration was hindered due to crossing hands, the likelihood to perceive a single tactile event increased. However, it remains to be further explored how increasing spatial separation between the crossed hands affects the intensity of illusory self-touch.

Furthermore, the follow up experiment (Experiment 3) revealed that not only crossing the hands over the midline, but also crossing them on one side of space (hemispace), increases the ratings of the self-touch illusion. The conflict between the somatotopical and external spatial frames of reference does not pertain to the fact that the hands are in their opposite sides of space with respect to the body midline, but it seems rather that crossing the hands per se is sufficient for enhancing the illusory self-touch. This can be linked to abundant literature on the use of different reference frames (body part rather than midline centred) for mapping tactile stimuli in healthy subjects, right brain damage patients with neglect and non-human primates [46], [47], [69], [70].

We also applied the proprioceptive judgment measure in our tactile RHI experiments. In previous studies on the tactile RHI, drift towards the rubber hand illusion was found to be greater after synchronous stroking [41], [43], [71]. We found a larger drift of synchronous tactile stimulation on the drift measure towards the rubber hand in Experiments 1 and 3 (and marginally in Experiment 2). However, the manipulation of hand posture did not influence proprioceptive judgments.

The absence of the posture manipulation effect on the proprioceptive drift can be due to the fact that the spatial separation between the receiving and administering hand was the same in the uncrossed and crossed postures. Also, as the hand drift is never complete (it ranges between 15–30% of the distance between the real and rubber hand [49]), there might exist an upper limit of the hand mislocalization, which might be reflected in our data. Our results could also be confounded by unbalanced male to female ratio across the experiments. In Experiment 2, we had a large majority of male subjects and in accordance with reported gender differences in proprioceptive sensitivity this may have affected our data; we note, however, that the existing findings on gender differences in proprioceptive abilities are rather sparse and inconsistent, as the superiority on the non-visual proprioceptive pointing tasks was evidenced for females [72] as well as for males [73]. Furthermore, we measured the felt location of the stroked hand, which was receiving the touch, but not the mislocalization of the stroking hand. A recent study on the tactile RHI found the proprioceptive drift of the stroking hand to be larger compared to the stroked hand, which is traditionally used for the measure of mislocalization [43]. Last, the absence of a postural modulation of the proprioceptive drift in Experiment 1 might be related to the sample size, it is possible that a larger sample size might have resulted in a significant crossed hands effect on proprioceptive mislocalization towards the rubber hand.

In Experiment 4 we investigated how crossing the hands influences the experience of illusory ownership and proprioceptive drift in the visual RHI. The participant's right hand was stroked in synchrony with the viewed rubber hand while his hands were uncrossed, crossed over his body midline or crossed in the right hemispace. Although the misalignments between the somatotopic and external reference frame were the same and the participant's hands were occluded from view in both the tactile and visual RHI versions, we found no additional effects of crossing the hands in the visual RHI. Our data suggest that visual capture of touch, due to high spatial resolution of visual information, provided a strong external space reference, into which the tactile stimulus was coded. By dominating the remapping process of tactile stimuli into the external reference frame, vision overrode the proprioceptive cues from actual hand position, so that the felt and seen locations of the tactile stimuli were matched.

When taking into account the existent studies on postural manipulations in the visual RHI, where observed illusory ownership decreased with larger visuo-proprioceptive mismatches between the real and rubber hand [32], [37], [38], our results might appear contradictory at first glance. However, importantly, although the position of the participant's arms varied throughout the three conditions, the handedness, orientation and distance between the participant's stroked and rubber hand was constant in all conditions. Although the reliance on the proprioceptive cues might be reduced due to the arms being crossed, the visuo-proprioceptive similarity between the hands themselves did not change. In this sense, the studies cannot be completely compared. However, recent findings by Cadieux, Whitworth and Shore [74] are relevant. Using the visual RHI paradigm, they showed that when hands were crossed over the midline, the proprioceptive drift, contrary to our findings, diminished as compared to uncrossed posture. They explain the reduction of proprioceptive drift as a consequence of impaired tactile, visual and proprioceptive signal integration due to crossed posture. However, it is not possible to compare results of Cadieux et al. with those from the present study, because they did not collect subjective questionnaire data and thus no information about how crossing the hands affected illusory hand ownership in their study is available.

In conclusion, the present study is the first to show that crossing the hands enhances illusory self-touch in the tactile RHI paradigm. The study also links the illusion to well-established knowledge of posture effects on proprioceptive coding. Crossing the hands is a powerful manipulation to maximise the misalignment of the somatotopic and external reference frames. As this postural manipulation induces strong tactile-proprioceptive conflict, it is observed as a deficit on certain tactile processing tasks, while in the context of the tactile RHI it leads to enhanced illusory self- touch. Crossing the hands implies re-weighting of tactile and proprioceptive signals, leading to enhanced probability that two, spatially separated, but temporally matched tactile stimuli are mapped to the same location in the peripersonal space, and thus perceived as self-touch.
